# Tell Me What You’ve Done, and I’ll Predict What You’ll Do: The Role of Motivation and Past Behavior in Exercise Adherence

**DOI:** 10.3390/healthcare13151879

**Published:** 2025-08-01

**Authors:** Luís Cid, Diogo Monteiro, Teresa Bento, Miguel Jacinto, Anabela Vitorino, Diogo S. Teixeira, Pedro Duarte-Mendes, Vasco Bastos, Nuno Couto

**Affiliations:** 1 Sport Sciences School of Rio Maior, Santarém Polytechnic University (ESDRM-IPSantarém), 2040-413 Rio Maior, Portugalteresabento@esdrm.ipsantarem.pt (T.B.); anabelav@esdrm.ipsantarem.pt (A.V.); 2 Research Centre in Sports Sciences, Health Sciences and Human Development (CIDESD), 5000-558 Vila Real, Portugal; diogo.monteiro@ipleiria.pt (D.M.); miguel.s.jacinto@ipleiria.pt (M.J.); 3 School of Education and Social Sciences (ESECS), Polytechnic University of Leiria, 2411-901 Leiria, Portugal; 4 Faculty of Physical Education and Sport, Lusófona University, 1749-024 Lisboa, Portugal; diogo.teixeira@ulusofona.pt (D.S.T.); vasco.bastos@ulusofona.pt (V.B.); 5 Research Center in Sport, Physical Education, and Exercise and Health (CIDEFES), 1749-024 Lisboa, Portugal; 6 Department of Sports and Well-Being, Polytechnic Institute of Castelo Branco, 6000-084 Castelo Branco, Portugal; pedromendes@ipcb.pt; 7 Sport Physical Activity and Health Research and Innovation Center (SPRINT), 6000-084 Castelo Branco, Portugal

**Keywords:** motivation, exercise, physical activity, behavior regulation, achievement goal theory, self-determination theory

## Abstract

**Introduction:** The main purpose of this study was to test a hierarchical model of motivation that integrates Achievement Goal Theory and Self-Determination Theory to explain and predict exercise adherence. **Method:** In total, 2180 exercisers (1020 female, 1160 male) aged between 18 and 60 years, from different gyms and health clubs, completed several scales validated in exercise settings, regarding perceived motivational climate, basic psychological need satisfaction, behavioral regulation, and exercise adherence. For the last measure, weekly computer access to a control system over a 6-month period before and after data collection was consulted. **Results:** Through structural equation models (SEM), it was verified that (1) task-involving climate positively predicted basic psychological needs. In turn, the satisfaction of these needs predicted autonomous motivation, which led to a positive prediction of adherence; (2) a small variation in exercise adherence was explained by the motivational model under analysis. Nevertheless, models significantly improved their analytical power when past adherence was inserted in the model increasing the explained variance in future behavior from 9.2% to 64%. **Conclusions:** In conclusion, autonomous motivation can predict people’s exercise adherence, and past behavior increases that predictive effect. The present study brings scientific evidence to the popular saying “*tell me what you’ve done and, and I’ll predict what you’ll do*”.

## 1. Introduction

Despite excessive exercise possibly also reflecting underlying psychological vulnerabilities [1,2,3], a large body of evidence has shown that adequate regular exercise has unquestionable physical, psychological, and social health benefits that can contribute significantly on reducing these indicators and enhancing general health and well-being in all age groups [4,5,6,7].

Globally, the prevalence of insufficient physical activity has increased over the past two decades, reaching 31.3% in 2022, up from 23.4% in 2000 and 26.4% in 2021 [8]. These trends are consistent with earlier findings by Hallal et al. [9] and Guthold et al. [10]. In Europe, recent data also highlights concerning levels of inactivity. According to special Euraborometer 525 [11], only 6% of Europeans report engaging in sport or physical activity regularly, continuing a decline observed in a previous survey [11,12,13,14].

In Europe, the most frequently reported barriers to physical activity are lack of time (41%) and lack of motivation or interest 25% [15,16]. For this matter, motivational approach research that allows a better understanding and reverses actual physical inactivity trends is warranted.

Achievement Goal Theory (AGT; Nicholls [17,18]) explains how individuals interpret and respond to achievement situations based on motivational climate and goal orientation. This theoretical framework distinguishes between task-involving climates, which emphasize commitment, effort, cooperation, and personal growth [19], and ego-involving climates, which prioritize outcomes and external validation [19,20]. Task-involving climates are positively associated with adaptive motivational patterns, well-being, and behavioral persistence, in contrast to ego-involving climates [21,22]. These climates are shaped by professionals (e.g., coaches, instructors) and perceived by participants (e.g., athletes, exercisers), influencing motivation and behavior in achievement contexts [21].

Another theoretical framework used on motivation subjects in several contexts is Self-Determination Theory (SDT: Deci and Ryan [23]; Ryan and Deci [24]), which describes behavioral regulation as a continuum which explains the internalization of behavior from a less self-determined form to a more self-determined form of motivation. SDT holds that behavior regulation is mediated through satisfaction in the following three basic psychological needs (BPN): autonomy, competence, and relatedness. Such needs are essential in psychological well-being, optimal function, as well as for motivation. In circumstances in which environments provide for satisfaction in BPN, individuals tend to internalize behavior as well as experience stronger self-determined forms of motivation [24].

Over the last few years, the inclusion of theoretical frameworks, such as AGT and SDT, has provided significant insight into sport and exercise behavior’s motivational mechanisms [25,26,27]. However, very little research has investigated their joint effect on exercise attendance, particularly in gym or health club settings.

AGT centers on the impact of the motivational climate, as mediated through significant others, for instance, exercise professionals, specialists, or coaches. Such climates, being either task-involving or ego-involving, impact exercising views of success and physical exercise. The diversification of the class schedule can affect these views, with a subsequent influence on motivation and behavior [28,29].

In parallel, SDT posits that the satisfaction of BPN plays a central role in fostering self-determined motivation. The integration of AGT and SDT is, therefore, particularly meaningful when considering the influence of motivational climate on BPN satisfaction or frustration. While research has begun to explore the positive association between task-involving climates and BPN satisfaction (e.g., Álvarez et al. [30]; Duda [19]; Markland and Tobin [31]), findings to date remain somewhat limited and primarily suggestive. Evidence from studies that have explicitly combined both theoretical perspectives indicates that task-involving climates positively predict BPN satisfaction [30,31,32], whereas ego-involving climates are negatively associated with BPN [20].

Furthermore, BPN satisfaction has been shown to mediate the relationship between motivational climate and autonomous forms of motivation, which in turn are robust predictors of pleasure, well-being, and sustained engagement in exercise and sport contexts [29,33,34]. These findings underscore the relevance of integrating AGT and SDT frameworks to better understand the complex motivational processes that underpin long-term exercise adherence in real-world fitness settings. According to the Hierarchical Model of Intrinsic and Extrinsic Motivation (HMIEM: Vallerand [35]), these findings support a causal pathway in which the motivational climate influences BPN satisfaction, which in turn fosters autonomous motivation, ultimately predicting exercise adherence. Although previous studies [33,36] have explored individual links of the HMIEM model, to our knowledge, no prior research has tested the full sequence from motivational climate to adherence using objective, longitudinal data in gym settings.

Considering the amount of evidence, it seems reasonable to assume that the perceived climate could influence BPN, which would regulate differentiated behavioral motivations with consequences to exercise adherence [29,33,34]. Some studies have reported intentions or subjective experience of exercise participation as criterion for future behavior [37], without objectively controlling maintenance (i.e., frequency). However, Hagger et al. [38] explained that past behavior plays an important role in future behavior, since it has a direct effect regardless of intentions. In addition, the inclusion of past behavior in motivational models typically attenuates the effect of intention on future behavior. This occurs because past behavior captures the influence of habitual patterns, environmental stability, and behavioral automaticity, which can drive future actions beyond the scope of current intentions [38]. Recently, a study conducted by Gomes et al. [39] with 454 gym and health club exercisers, using computer recordings to measure three months of past frequency, showed that past behavior explained only 10% of future behavior. However, these authors only considered three months of exercise practice, which may have influenced their results [40]. Three months of exercise records may be too short for explaining exercise adherence, since according to the Transtheoric Model [41], namely the maintenance phase, individuals need to exercise regularly for at least six months so that the behavior can me maintained and the risk of dropout diminished. In addition, the dropout exercise curve [42,43] explains that exercise participation decreases abruptly by about 50% in the first six months, stabilizing from this period beyond. Other recent studies addressed this issue, but they did not monitor the frequency continuously (i.e., weekly), only the total number of entries on the gym, so they do not ensure that the exercisers practice was regular over time or that there were no periods of dropout [36,44]. To overcome these limitations, the present study operationalized continuous adherence as the average weekly attendance over a six-month period, using objective data from the gyms’ electronic access systems.

Therefore, this study aimed to analyze AGT and SDT in one motivational model to predict exercise adherence in gym and health club exercisers. For this matter, the following causal model will be tested: motivational climate (task-involving climate) → psychological mediators (BPN satisfaction) → motivation (autonomous motivation) → exercise adherence (after 6 months). Afterwards, mediation effect of the past behavior (previous 6 months) in future adherence’s (6 months after) prediction will be examined. Based on this rational, the following research hypothesis were formulated.

**H1.**  *Task-involving climate is positively associated with BPN satisfaction*.

**H2.**  *BPN satisfaction is positively associated with autonomous motivation.*

**H3.**  *Autonomous motivation is positively associated with future exercise adherence (after six months)*.

**H4.**  *Past behavior mediates the relationship between autonomous motivation and future exercise adherence.*

## 2. Method

### 2.1. Study Design

This study followed a quantitative, non-experimental, observational, and partially longitudinal design. Its main objective was to test a causal motivational model, based on theoretical principles of AGT and SDT theories to predict exercise adherence in a fitness context.

### 2.2. Participants

A total of 2180 subjects (1020 females, 1160 males) aged between 18 and 60 years (M = 32.4; SD = 10.4) were recruited using a convenience method through direct contact in fitness centers and online fitness communities. Inclusion criteria required participants to be aged 18 or older, currently engaged in regular exercise in gyms or health clubs, and to have between 6 and 240 months of exercise experience (M = 27.1; SD = 29.1).

Participants performed various type of activities (strength training; group classes; cardio activities—mixture of cardio and strength training activities). Weekly gym attendance ranged from 1 to 6 session per week (M = 3.1; SD = 1.1), and the average training duration was 5.0 ± 2.4 h per week (ranging from 1 to 15 h).

### 2.3. Procedures

Initially, gym and health club managers were contacted for permission to collect data and for access to participants’ attendance records in computerized form. Researchers randomly stopped participants on afternoons in the reception area, predominantly during late-afternoon sessions during weekdays. After being briefly explained the purpose of the study, permission for access to their attendance data was granted, and participants responded to a paper-based survey in standard conditions.

The study was approved for ethical clearance by the University Ethics Committee. Informed consent forms were signed for all participants, who were also informed about the confidentiality of their information. No financial remuneration nor material rewards were provided. It took between 15 min and 20 min for participants to complete the survey.

The following phases were adopted:

(1) Pre-selection (weeks 1 and 2): prior to data collection, all participants with less than six months of practice and/or who had more than one episode of withdrawal (at least a month) were excluded.
(2) Gym attendance verification (weeks 4 and 5): Participants were selected, which matches the number of subjects who had a weekly frequency (i.e., at least one time a week) in the six months prior to data collection and in the following six months. Regarding gym attendance criteria. Given the specificity of exercise practice, it is very common for people to have several withdrawal episodes, especially in summer holidays. Therefore, only one episode of withdrawal (i.e., with no weekly attendance) was allowed. These criteria guarantee people exercise continuously for an extended period.
(3) Final verification (weeks 26 and 27): Six months after data collection, the same criteria were applied to identify the participants who kept the minimum weekly attendance in the last six months and had a single episode of withdrawal. Due to the nature of common conflicts in exercise practice, especially during periods such as summer vacation, it is acceptable to consider short episodes of withdrawal as part of an ongoing pattern of practice [45]. Studies indicate that temporary interruptions do not necessarily compromise exercise adherence, as long as the minimum frequency (e.g., once a week) is maintained most of the time [46].

### 2.4. Instruments

The Perceived Motivational Climate in Exercise Questionnaire (PMCEQ: Thomas and Barron [47]; Portuguese version: Cid et al. [48]) consists of a 10-item assessing task and ego involving climates. Responses use a 5-point Likert scale (1 = “Totally disagree” to 5 = Totally Agree). Only the task-involving subscale was used in the current study.

The Basic Psychological Needs in Exercise Scale (BPNES: Vlachopoulos and Michailidou [49], 2006; Portuguese version: Moutão et al. [50]) was used. This 12-item covering measurement encompasses autonomy, competence, and relatedness. Responses use a 5-point Likert (1 = “Totally disagree” to 5 = “Totally agree”). A composite factor, validated by Cid et al. [51], was used as in previous research [49,51].

The Behavioral Regulation in Exercise Questionnaire (BREQ-3: Cid et al. [52]) was used. This measure emerges from the original version (BREQ-2: Markland and Tobin [53]) and the Portuguese version (Cid et al. [54]) to which the “integrated regulation” scale developed by Wilson et al. [55] was added. This 24-item measurement encompasses the behavioral regulations (amotivation, external, introjected, identified, integrated, intrinsic). Responses are rated on a 5-point Likert scale (0 = “Not true for me” to 4 = “Very true for me”). A composite score for autonomous motivation, validated by Cid et al. [54], was used [56].

Adherence was measured using electronic access control systems at the participating gyms. Attendance was automatically recorded via individual membership card check-ins. The study considered the average weekly attendance during the six months prior to data collection (past adherence) and the six months following data collection (future adherence). This method is consistent with previous studies (e.g., Vlachopoulos and Neikou [40]; Rodrigues et al. [36,44]).

### 2.5. Statistical Analysis

Descriptive statistics (means and standard deviations) and bivariate correlations were run with IBM SPSS Statistics v27.0.

A two-step maximum likelihood (ML) method following Kline’s [57] recommendations were adopted using AMOS 23.0. Confirmatory Factor Analysis (CFA) was used to analyze the models’ psychometric properties. Composite reliability via Raykov formula (1997) was assessed accepting values ≥ 0.6 as suggested by Hair et al. [58]. Additionally, convergent and discriminant validity where evaluated via average variance extracted (AVE ≥ 0.50) and the square of correlations between factors (r^2^ < VEM), respectively, following several recommendations (e.g., Hair et al. [58]).

In the second step, Structural Equation Modeling (SEM) to test the proposed relationships among studied variables was performed. Following the recommendations of Cid et al. [59], data was analyzed according to several authors [57,58,60], and the following goodness-of-fit indexes were used for both steps (CFA and SEM): Standardized Root Mean Square Residual (SRMR), Comparative Fit Index (CFI), Non-Normed Fit Index (NNFI), Root Mean Square Error of Approximation (RMSEA), and its respective confidence interval (RMSEA 90% CI). The subsequent cut-off values were adopted: SRMR ≤ 0.08, CFI, TLI ≥ 0.90, and RMSEA ≤ 0.08 [58,60].

A bootstrap resampling approach (1000 samples) was taken in an attempt to approximate the direct and indirect effects during the mediation analysis, in accordance with Hayes’ [61] guidelines. Indirect effects were significant when the 95% confidence interval excluded zero (*p* ≤ 0.05).

## 3. Results

### 3.1. Preliminary Analysis

Data revealed that there were no missing values or outliers (univariate and multivariate) and no deviation from univariate normality (skewness values varied between −2 and +2 and kurtosis values varied between −7 and +7). However, Mardia’s coefficient of multivariate kurtosis was above 5 as the recommended value. Therefore, a Bollen Stine bootstrap of 2000 samples was performed, as suggested by Nevitt and Hancock [62]. The sample size calculations were performed a priori using the Soper Calculator [63] for structural equation modeling. The objective was to determine the optimal sample size required to effectively test the hypothesized model. Factors such as the anticipated effect size (0.1) desired statistical power (*p* = 0.08), significance level (*p* = 0.05), number of latent variables (3), and observed variables (22) were considered. By utilizing the Soper Calculator, we ensured that the sample size was adequate to yield reliable and meaningful results, with a minimum sample size of 1258 participants to detect the effect, which was respected in the present study.

Finally, possible collinearity issues were analyzed through the Variance Inflation Factor (VIF). Results showed values < 10, meaning that there are favorable conditions to accomplish regression analysis, as recommended by Hair et al. [58].

In Table 1, descriptive statistics are exhibited, with bivariate correlations and composite reliability and convergent validity of variables under analysis. The results displayed high means in all variables (above the midpoint), as well as positive and significant associations among all constructs, except for task-involving climate on past and future adherence and adjusted values of composite reliability and convergent validity.

### 3.2. Measurement Model (CFA)

Regarding CFA models (Models 1 and 2), both fit the data. Specifically, Models 1 and 2 present the following goodness-of-fit indexes: χ^2^ = 48.199 (49); B-S *p* = 0.301; TLI = 0.960; CFI = 0.972; RMSEA = 0.033 (90%CI = 0.000, 0.061); SRMR = 0.0489; χ^2^ = 58.695 (57); B-S *p* = 0.260; TLI = 0.967; CFI = 0.977; RMSEA = 0.036 (90%CI = 0.000, 0.060); SRMR = 0.0489, respectively. Convergent validity and discriminant validity were also achieved.

### 3.3. Structural Equation Model (SEM)

**Figure 1:**
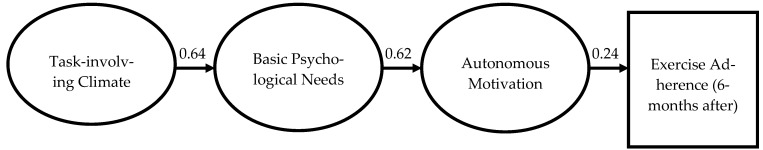
Individual parameters standardized from Model 1.

Looking at Figure 1, the model indicates that task-involving motivational climate is a predictor of BPN satisfaction (β = 0.64; [0.453, 0.826]; R^2^ = 0.41). BPN predict significantly positive autonomous motivation (β = 0.62; [0.344, 0.811]; R^2^ = 0.38). In turn, autonomous motivation is a positive and significant predictor of future exercise adherence (β = 0.24; [0.099, 0.399]; R^2^ = 0.06). The standardized indirect effects showed that a task-involving climate positively predicted self-determined motivation (β = 0.40; [0.216, 0.549]), through need satisfaction. Task-involving climate through autonomous motivation (β = 0.10; [0.035, 0.207]) and basic psychological needs satisfaction through autonomous motivation (β = 0.15; [0.053, 0.313]) positively predicted forthcoming exercise adherence. The model presented the following goodness-of-fit indexes: χ^2^ = 51.822 (52); B-S *p* = *0*.294; TLI = 0.961; CFI = 0.970; RMSEA= 0.033 (90%CI = 0.000, 0.059); SRMR = 0.0511. The model accounts for 6% of the variance in future exercise adherence (R^2^ = 0.06), with this variance being predominantly explained by indirect effects. Specifically, both a task-involving motivational climate and the satisfaction of basic psychological needs exert an indirect influence on adherence through their positive impact on autonomous motivation. The total standardized indirect effects represent approximately 0.25 of the total effect, while the direct effect of autonomous motivation on adherence is 0.24. These findings indicate that the explanatory power of the model is primarily driven by indirect pathways, underscoring the central mediating role of autonomous motivation in shaping future exercise adherence.

**Figure 2:**
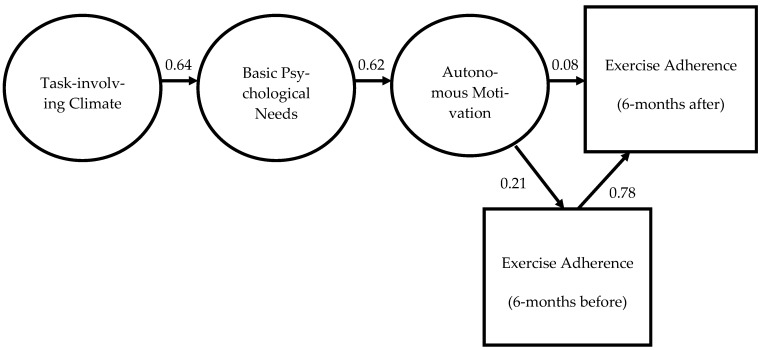
Individual parameters standardized from Model 2.

In Figure 2, the model that addresses the mediation role of past 6 months of exercise behavior in the prediction of future practice (6 months after) is presented. This model presented the following values: χ^2^ = 64.766 (62); B-S *p* = 0.248; TLI = 0.967; CFI = 0.975; RMSEA = 0.035 (90% CI = 0.000, 0.059); SRMR = 0.0526. The results show that past adherence has a mediating effect between autonomous motivation and future adherence, exhibiting as the strongest predictor in the model (β = 0.78 [0.702, 0.836]; R^2^ = 0.64). Moreover, it is possible to confirm mediation roles by analyzing the indirect effects between parameters. The direct effect is not significant (β = 0.08 [−0.010, 0.174]), thus autonomous motivation has a significant indirect effect on future adherence (β = 0.16 [0.046, 0.276]) through the mediator variable (i.e., past adherence). Therefore, results confirm the mediation effect of past behavior. Task-involving climate (β = 0.08 [0.023, 0.209]) and basic psychological needs satisfaction (β = 0.13 [0.036, 0.270]) positively predicted past adherence (R^2^ = 0.04) through autonomous motivation. Considering the present results, the formulated hypothesis can be accepted.

## 4. Discussion

Grounded in AGT and SDT, this study employed the HMIEM framework [35] to examine the motivational determinants of exercise adherence in gyms and health clubs, focusing on the mediating role of autonomous motivation between past and future behavior.

As illustrated in Figure 1, a task-involving climate positively predicted basic psychological need (BPN) satisfaction. In turn, BPN satisfaction was associated with more self-determined forms of motivation, which subsequently predicted future exercise adherence. These findings underscore the value of the “bright side” of motivation [36,44].

According to AGT and SDT theories [64,65,66,67,68], motivation is mediated by the satisfaction of BPN, influencing controlled and autonomous regulation of behavior, and exercise is more persistent in activities when motivation is self-determined. These theories suggest that context supports intrinsic motivation, which is linked to achievement and well-being. Task-involving climates and autonomy-supportive contexts promote adaptive motivational patterns, BPN satisfaction, and sustained engagement [21,29,67].

This is a critical point, as intrinsic motivation has consistently been identified as one of the most significant predictors of long-term engagement in physical activity [68], a finding supported by a wide body of empirical research [29,33,34]. Beyond its theoretical grounding, the results of the present study are also empirically corroborated. Several authors have demonstrated the interplay between constructs from AGT, notably motivational climate, and those from SDT, such as BPN and behavioral regulation [69,70].

The impact of these constructs on behavioral outcomes has been widely documented. For example, task-involving climates and greater BPN satisfaction have been associated with increased commitment and stronger intentions to engage in physical activity [71,72], lower dropout rates in sport [73], greater subjective vitality [74], more positive effects and participation [75], favorable attitudes toward physical activity [76], increased enjoyment and reduced boredom [30,44], greater persistence [36], stronger intentions to continue [54], and enhanced perceived effort [32]. Importantly, the present study found significant positive indirect effects between basic psychological needs satisfaction and exercise adherence. This is consistent with previous research indicating that BPN, particularly competence, play a critical role in influencing physical activity behaviors, whether assessed through self-reported [75] or observational methods [36,49,76]. These findings reinforce the importance of psychological need satisfaction as a central determinant of motivational outcomes in exercise contexts.

Furthermore, autonomous motivation was shown to have a significant and positive effect on future adherence. These findings are in line with a systematic review by Rodrigues et al. [29], which examined 35 studies focused on exercise adherence and persistence, concluding that autonomous motivation is a strong and consistent predictor of sustained participation in gym and fitness settings. Additionally, our results support the notion that autonomous motivation plays a key role in the formation of future exercise habits. As highlighted by Lally and Gardner [77], self-determined motivation interacts with the frequency of behavior to predict levels of automaticity, suggesting that higher autonomous motivation facilitates the internalization and habitualization of exercise behaviors over time, and self-determined regulation compared to non-self-determined or controlled motivation is more associated with behavior habit.

With respect to the second objective of this study, examining the mediating role of past behavior and future behavior through autonomous motivation, the findings indicate that, in the absence of autonomous motivation, basic psychological needs (BPN) exert a direct, significant, and positive influence on future adherence. However, when autonomous motivation is included in the model, while maintaining the direct path from BPN to adherence, the direct effect remains significant but is attenuated, and the indirect effect via autonomous motivation becomes nonsignificant. According to the criteria outlined by Hagger and Chatzisarantis [21] and Hair et al. [58], these results do not provide support for either full or partial mediation. In this context, BPN appears to exert a direct influence on adherence, independent of behavioral regulation. One possible explanation lies in the specificity of the gym and health club environment, where particular needs, especially competence, may exert a more immediate influence. As noted by Ryan and Deci [68], competence satisfaction relates to the intrinsic experience of mastery and accomplishment, which individuals naturally seek for their own well-being. Similarly, Vlachopoulos and Neikou [40] emphasize that the need to feel effective and to engage in situations that allow the expression of personal abilities is particularly salient in fitness contexts. Thus, achievement-oriented goals may make BPN, especially competence, a strong and direct predictor of exercise maintenance in these settings [40,78]. Regarding the explanatory power of the model, the total effects in Model 2 accounted for only 9.2% of the variance in future adherence, underscoring the need for further research into the role of past behavior as a mediating variable (see Figure 2). Despite the modest explained variance, the model exhibited good fit indices, and all criteria for model adequacy were satisfied [58,60].

Importantly, the results provide clear evidence that past adherence—specifically behavior over the previous six months—mediates the relationship between autonomous motivation and future exercise behavior. In fact, past behavior emerged as the strongest predictor in the model. Mediation can be further confirmed by analyzing the indirect effects (see Table 2); when the indirect path from autonomous motivation to future adherence through past behavior is significant and the direct path is not, a mediation effect is established. The results also revealed a substantial increase in the explained variance for future behavior when past adherence was included, rising to 64%.

This is consistent with theoretical and empirical literature, which emphasizes that the development of more complex health-related habits, such as regular physical activity, is shaped by multiple factors, including self-regulatory processes [79] and behavioral history [80]. Hagger [81] notes that repeated performance of a behavior contributes to habit formation, facilitating a transition from intentional to automatic control. This mechanism has been well documented across several life domains, including physical activity [38,81,82,83].

Recent evidence supports this point, showing that past behavior frequently acts as a dominant mediator that absorbs the effects of motivational constructs on future adherence [44]. In this way, when past behavior is included in the model, the direct effect of autonomous motivation tends to become non-significant, and its influence is mostly driven through established behavioral patterns. These results suggest that past behavior could be a proxy for habit strength, capturing elements such as behavioral automaticity and environmental stability that motivational constructs alone may not fully account for [77].

Several factors contribute to the process by which an individual transforms a behavior into a habit, including the strength of intention, the perceived complexity of the behavior, and the application of self-regulatory strategies. For instance, in a longitudinal study conducted by Kaushal and Rhodes [84] involving first-time gym attendees over a 12-week period, findings indicated that participants who exercised at least four times per week during the first six weeks were significantly more likely to report habit formation. Similarly, Armitage [85], in a study of gym attendance over the same timeframe, found that attendance patterns during the initial five weeks were predictive of subsequent adherence, whereas attendance from week six onwards was not highlighting the crucial role of early behavior in habit development. Notably, both studies draw their conclusions primarily from the analysis of past behavior.

Despite these insights, relatively few long-term studies have explored the determinants that facilitate or hinder habit formation, underscoring the need for further empirical investigation in this domain. The findings of the present study align with recent research by Hagger [81], reinforcing the pivotal role of past behavior as the most reliable predictor of future behavior in the context of physical activity. This evidence supports the notion that consistent engagement in exercise over time contributes to the development of automaticity, thereby increasing the likelihood of sustained participation. From a practical standpoint, these findings may provide valuable guidance for organizations and practitioners seeking to design effective interventions aimed at increasing physical activity levels through habit formation strategies.

## 5. Limitations and Agenda for the Future

Although the present study contributes to understanding exercise adherence, it has some limitations that should be considered. Autonomy, competence, and relatedness, as well as identified and intrinsic motivation, were combined as a composite factor, BPN, and autonomous motivation, respectively. This was carried out to reduce the number of parameters to be estimated and to avoid collinearity considering sample size. Although several studies in exercise context have used similar procedures (e.g., Klain et al., [28]), in future studies, it is important to acknowledge that this approach may overlook the distinct effects of each BPN, specifically competence, which has often been identified as a strong predictor of behavioral adherence [33]. Forthcoming research in this field could examine these constructs independently to better understand their specific contributions. Additionally, this study did not analyze group differences (e.g., gender, activity type, or experience level). It is possible that key determinants, such as task-involving climate, BPN satisfaction, and adherence, may operate differently across these groups. Future research should consider multigroup analyzes to explore such variations and increase the generalizability of findings. Still, results in this study point out the fact that past behavior appears to be the strongest character in decision-making exercise as a habit [79,81].

## 6. Conclusions

In sum, the findings underscore the critical role of fostering a task-involving motivational climate, one that prioritizes effort, personal growth, and learning over competitive outcomes, to enhance the satisfaction of BPN. In turn, its satisfaction promotes more self-determined forms of motivation, which are essential for sustaining long-term engagement in exercise. Notably, the mediation analysis revealed that individuals with a consistent exercise routine over the past six months are 64% more likely to maintain their behavior, emphasizing the powerful reinforcing effect of past behavior in habit formation. From a theoretical perspective, this study reinforces key tenets of Self-Determination Theory (SDT), demonstrating the mediating role of BPN satisfaction and autonomous motivation in predicting exercise adherence. These insights have important practical implications; fitness professionals should be trained to cultivate task-involving environments that support BPN and foster autonomous motivation. Additionally, implementing regular assessments of client motivation may enable more personalized programming and timely interventions, particularly for individuals at greater risk of dropout.

## Figures and Tables

**Table 1:** Mean, standard deviations, and bivariate correlational of study variables.

	M ± SD	MC	BPN	AM	PA	FA	CR	AVE
Task-Involving Climate	4.00 ± 0.50	-					0.65	0.72
Basic Psychological Needs	4.02 ± 0.38	0.40 **	-				0.80	0.63
Autonomous Motivation	3.43 ± 0.49	0.27 **	0.37 **	-			0.63	0.55
Past Adherence	61.1 ± 28.7	−0.01	0.13 *	0.14 *	-		-	0.96
Future Adherence	54.1 ± 25.2	0.03	0.21 **	0.13 *	0.80 **	-	-	0.94

Note: M = mean, SD = standard deviation, MC = task climate; BPN = basic psychological needs satisfaction, AM = autonomous motivation; PA = past adherence, FA = future adherence; CR = composite reliability, AVE = average variance extracted, * *p* < 0.05; ** *p* < 0.01.

**Table 2:** Standardized effects (total, direct, and indirect effects).

Parameter	Total Effects	Direct Effects	Indirect Effects
Task-Involving Climate → BPN	0.64 *	0.64 *	-
Task-Involving Climate → Autonomous Motivation	0.39 *	-	0.39 *
Task-Involving Climate → Past Adherence	0.08	-	0.08
Task-Involving Climate → Future Adherence	0.09	-	0.09
BPN → Autonomous Motivation	0.62 *	0.62 *	-
BPN → Past Adherence	0.13 *	-	0.13 *
BPN → Future Adherence	0.15 *	-	0.15 *
Autonomous Motivation → Past Adherence	0.21 *	0.21 *	-
Autonomous Motivation → Future Adherence	0.24 *	0.08	0.16 *
Past Adherence → Future Adherence	0.78 *	0.78 *	-

Note: * significance at *p* < 0.05 (z scores ≥ 1.96).

## Data Availability

Data is available on request from the authors.
